# Comparative Analysis of Machine Learning Techniques for Water Consumption Prediction: A Case Study from Kocaeli Province

**DOI:** 10.3390/s24175846

**Published:** 2024-09-09

**Authors:** Kasim Görenekli, Ali Gülbağ

**Affiliations:** Faculty of Computer and Information Sciences, Sakarya University, Sakarya 54050, Turkey; agulbag@sakarya.edu.tr

**Keywords:** COVID-19 impact, gradient boosting machines, machine learning, random forest, water consumption prediction

## Abstract

This study presents a comparative analysis of various Machine Learning (ML) techniques for predicting water consumption using a comprehensive dataset from Kocaeli Province, Turkey. Accurate prediction of water consumption is crucial for effective water resource management and planning, especially considering the significant impact of the COVID-19 pandemic on water usage patterns. A total of four ML models, Artificial Neural Networks (ANN), Random Forest (RF), Support Vector Machines (SVM), and Gradient Boosting Machines (GBM), were evaluated. Additionally, optimization techniques such as Particle Swarm Optimization (PSO) and the Second-Order Optimization (SOO) Levenberg–Marquardt (LM) algorithm were employed to enhance the performance of the ML models. These models incorporate historical data from previous months to enhance model accuracy and generalizability, allowing for robust predictions that account for both short-term fluctuations and long-term trends. The performance of each model was assessed using cross-validation. The R^2^ and correlation values obtained in this study for the best-performing models are highlighted in the results section. For instance, the GBM model achieved an R^2^ value of 0.881, indicating a strong capability in capturing the underlying patterns in the data. This study is one of the first to conduct a comprehensive analysis of water consumption prediction using machine learning algorithms on a large-scale dataset of 5000 subscribers, including the unique conditions imposed by the COVID-19 pandemic. The results highlight the strengths and limitations of each technique, providing insights into their applicability for water consumption prediction. This study aims to enhance the understanding of ML applications in water management and offers practical recommendations for future research and implementation.

## 1. Introduction

- **Research Background**

Water scarcity is a critical global issue, with 25 countries facing extremely high water stress and approximately one-quarter of the world’s population affected [1]. Nations like Bahrain, Kuwait, and Israel experience severe shortages, while countries such as Mexico, Spain, and Turkey face high stress levels. Even countries with medium-high stress, like China and the United States, are not immune to this crisis. This global challenge is exacerbated by population growth, urbanization, and climate change, straining limited freshwater resources and intensifying competition among sectors [2]. Turkey, ranking 39th globally, exemplifies this challenge; its annual per capita water supply is projected to decrease from 1.365 to 1.120 cubic meters by 2030, highlighting the urgent need for improved water management and conservation efforts worldwide [3]. This study aims to address these challenges by leveraging advanced machine learning techniques to improve water consumption prediction, ultimately contributing to more effective water resource management in Turkey and similar regions facing water stress. Turkey faces significant challenges in water management. The country has a high rate of water leakage, with an estimated 37% of water lost through leaks and unauthorized usage [4]. Additionally, Turkey’s water availability per capita is below the global average, emphasizing the need for efficient water resource management and accurate consumption predictions [5].

- **Importance of Accurate Water Consumption Prediction**

**Figure 1:**
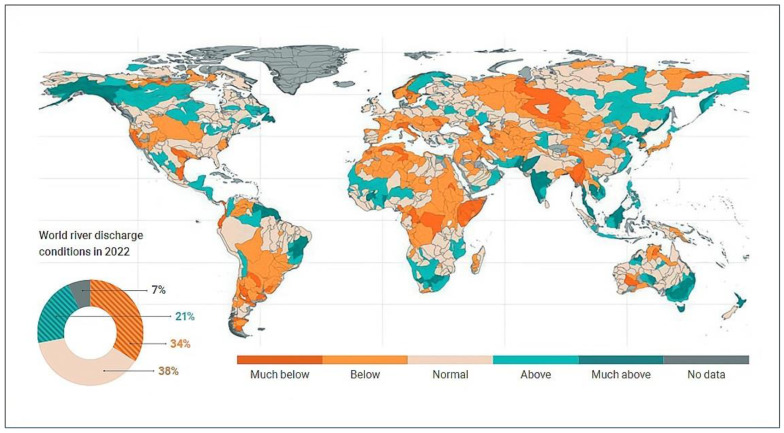
Mean River Discharge for the Year 2022 Compared to the Period 1991–2020 [6].

Accurate prediction of water consumption is crucial for effective water resource management and planning, especially considering the significant impact of the COVID-19 pandemic on water usage patterns. These data underscore the importance of accurate water consumption prediction models in addressing changing water availability patterns and their effects on agriculture, navigation, energy production, and water supply. Figure 1 illustrates global river discharge anomalies in 2022 compared to the 1991–2020 baseline for basins larger than 10.000 km^2^, highlighting significant deviations from normal conditions in over 50% of the global catchment area. Predominantly lower-than-normal discharge was observed, with notable impacts in South America, particularly the La Plata river basin.

- **Advantages of Machine Learning Methods**

Machine Learning (ML) techniques have emerged as powerful tools for predictive modeling due to their ability to handle large datasets and capture complex patterns. Traditional statistical methods, while useful, often fall short in dealing with nonlinear relationships and high-dimensional data. ML techniques such as Artificial Neural Networks (ANN) [7], Random Forest (RF) [8], Support Vector Machines (SVM) [9], and Gradient Boosting Machines (GBM) [10] offer improved accuracy and robustness in predictive tasks. Furthermore, ref. [11] demonstrated the effectiveness of machine-based statistical learning techniques in predicting residential water demand, highlighting their ability to capture complex patterns in water usage data. Ref. [12] conducted a comprehensive analysis and uncertainty assessment of various ML models for water quality prediction in Mirpurkhas, Sindh, Pakistan, demonstrating the versatility of these techniques in addressing different aspects of water resource management. Additionally, ref. [13] developed a deep learning-based prediction model for water consumption at the household level, showcasing the potential of deep learning techniques to capture intricate patterns in residential water usage and contribute to more accurate and granular predictions.

- **Optimization Methods**

In addition to the machine learning methods, this study also employs optimization techniques such as Particle Swarm Optimization (PSO) and the Levenberg–Marquardt (LM) algorithm. PSO is used to optimize the parameters of the machine learning models, enhancing their performance in predictive tasks. The LM algorithm, based on second-order derivatives, provides more accurate and faster convergence in certain optimization problems.

- **Purpose of the Research**

This study aims to conduct a comparative analysis of various ML techniques for predicting water consumption using a dataset from Kocaeli Province, Turkey. The focus is on evaluating the performance of different models in terms of accuracy, efficiency, and practical applicability. By analyzing the strengths and limitations of each technique, this research seeks to provide insights into the most effective approaches for water consumption prediction and contribute to the body of knowledge in water resource management. This study evaluates four machine learning models—ANN, RF, SVM, and GBM—alongside two optimization techniques, PSO and the LM algorithm, which are used to enhance model performance. This comprehensive analysis highlights the strengths and limitations of each approach, offering practical recommendations for selecting the most appropriate model based on specific needs and conditions.

## 2. Literature Review

Several studies have examined the use of ML techniques in predicting water consumption, emphasizing the importance of climatic factors and the need for comprehensive approaches [14,15,16]. These studies highlight the critical role of accurate water consumption prediction in effective water resource management. Our study builds upon this foundation by incorporating a comprehensive dataset that includes the impact of the COVID-19 pandemic, providing novel insights into water consumption patterns under unprecedented circumstances [17].

Advanced ML techniques, such as deep learning and ensemble learning methods, have shown superior performance in capturing temporal dependencies in water usage data. For example, ref. [9] demonstrated the effectiveness of these techniques in predicting residential water demand, highlighting their ability to capture complex patterns in water usage data. Ref. [18] explored the application of various short-term water demand forecasting models in Korea, utilizing real-time data collected through a Smart Water Grid (SWG) system. This study emphasized the advantages of real-time data in improving prediction accuracy. Ref. [19] developed an ANN model for both water quality and water consumption prediction, showcasing the versatility of ANN in handling different aspects of water resource management.

Data-driven approaches are increasingly used in water resource management, addressing challenges such as climate change and ecosystem destruction [20]. These approaches provide valuable insights into the impact of environmental changes on water resources.

Ref. [21] provided a review of AI applications in water consumption assessment, underscoring the advancements and future possibilities. This review highlighted the potential of AI in enhancing water resource management practices. ML techniques have been applied to related areas of water resource management, including rainfall prediction models and predicting drinking water potability [22,23]. Ref. [24] provided a review of water demand prediction methods, emphasizing the need for region-specific model selection. This aligns with our approach of comparing various machine learning techniques for Kocaeli Province, ensuring that the models are tailored to the specific conditions of the region.

Recent advancements include the use of deep learning methods, such as Long Short-Term Memory (LSTM) networks, which have shown superior performance in capturing temporal dependencies. Ref. [25] discussed the potential of data-driven modeling approaches in hydrology, highlighting their effectiveness in predicting hydrological patterns. Ref. [26] reviewed soft computing methods for water demand forecasting, emphasizing their advantages in handling complex and nonlinear data.

The implementation of smart meters has enhanced the ability to collect and analyze detailed water consumption data. Ref. [27] demonstrated the use of smart meters to learn water customer behavior, highlighting the benefits of detailed data collection in improving prediction accuracy.

Comparative studies have demonstrated the effectiveness of different ML techniques in various domains. For instance, in energy consumption prediction, deep learning models like LSTM have outperformed traditional models such as ARIMA [28]. This demonstrates the advantages of deep learning models in capturing complex temporal patterns. In the field of finance, ensemble methods have been successful in improving prediction accuracy. Ref. [29] explored the use of fuzzy logic in water demand forecasting for Dubai City. Ref. [30] presented a real-time data analysis platform for short-term water consumption forecasting using ML techniques, showcasing the benefits of real-time data analysis in improving prediction accuracy.

Spatio-temporal modeling techniques have been utilized in environmental studies to analyze complex datasets. For instance, ref. [31] employed spatio-temporal modeling to study particulate matter concentrations using satellite-derived aerosol optical depth over the coastal region of Chennai in India, demonstrating the effectiveness of spatio-temporal models in environmental studies.

While extensive research has been conducted on water consumption prediction, there is a noticeable gap in comprehensive comparative studies focusing on multiple ML techniques applied to a single dataset. Moreover, regional studies within Turkey, particularly involving detailed datasets like that from Kocaeli Province, are limited. This study aims to fill these gaps by providing a thorough comparison of various ML models on a consistent dataset, offering valuable insights for both academic research and practical applications.

Unlike previous studies, our research uniquely integrates a large-scale dataset of 5000 subscribers, incorporates the impact of the COVID-19 pandemic, and employs multiple ML techniques to provide a comprehensive analysis. This approach not only enhances the understanding of ML applications in water management but also offers practical recommendations for future research and implementation.

## 3. Materials and Methods

### 3.1. Dataset Description

This study employed a comprehensive dataset from the Kocaeli Province, Turkey, and covers a comprehensive range of data points crucial for water consumption prediction. This dataset includes water consumption records for 5000 subscribers selected from a total of over 800,000, based on the criterion that they did not change their subscription during the 80-month period from January 2016 to August 2022. The subscribers were categorized into three types: residential (3447), commercial (1422), and official (131). The dataset includes various features that potentially influence water consumption.

Daily weather data between January 2016 and August 2022 were collected by the Meteorology Directorate of Kocaeli, Kocaeli, Turkey, from 20 locations covering the whole of city via sensors, including parameters such as rainfall, sunshine duration, temperatures, humidity, and wind speed. Notably, occasional disruptions in data collection occurred due to extreme weather conditions or technical issues like power outages. In instances where a particular station was non-functional, data from the nearest operational stations were used to interpolate the missing values, ensuring continuity in the dataset while maintaining the integrity of the weather information used in the analysis.

For our analysis, we calculated monthly averages from this daily data. The dataset encompasses a geographical area of Kocaeli Province, which covers approximately 3.400 square kilometers [32]. Weather data were collected from 20 sensor locations strategically distributed across the province to ensure comprehensive coverage. The spatial resolution of the data collected is based on these sensor locations, allowing for localized weather influences to be captured in the analysis.

Water consumption data, on the other hand, were collected monthly through manual readings of water meters by utility staff. This approach allows us to align the temporal resolution of our weather and consumption data on a monthly basis, which is the forecasting period for our prediction models.

Temporal data included information on weekends, holidays, and the impact of the COVID-19 pandemic, categorized into pre-pandemic, during-pandemic, and post-pandemic periods. Subscriber information included household size and subscription type, and historical consumption data covered the previous four months’ usage, abbreviated as “prev4Month” (which represents the average consumption in tons over the last four months). Feature selection was performed using correlation analysis and feature importance ranking. Based on these analyses, the most relevant features were identified: precipitation, wind speed, sunshine duration, max humidity, min humidity, max temperature, min temperature, weekends, holidays, household size, pandemic period (COVID-19), and prev4Month. This feature selection process ensures that our models are built on the most informative variables, balancing predictive power with model simplicity. These features were chosen due to their significant impact on water consumption patterns, as identified in our analysis and previous studies [11].

The dataset includes comprehensive water consumption records for commercial, official, and residential subscribers. Sample data tables for each subscriber type are provided in Appendix A, illustrating the key features and data structure used in our analysis.

This approach ensures that the features listed in Table 1 are among the most influential for water consumption prediction, as identified through our analysis and supported by previous studies. This aligns with the feedback we received, which emphasized the need to clarify the importance levels of these features.

Table 1 provides a summary of the dataset inputs, highlighting the various types of data used in this study and their descriptions.

**Figure 2:**
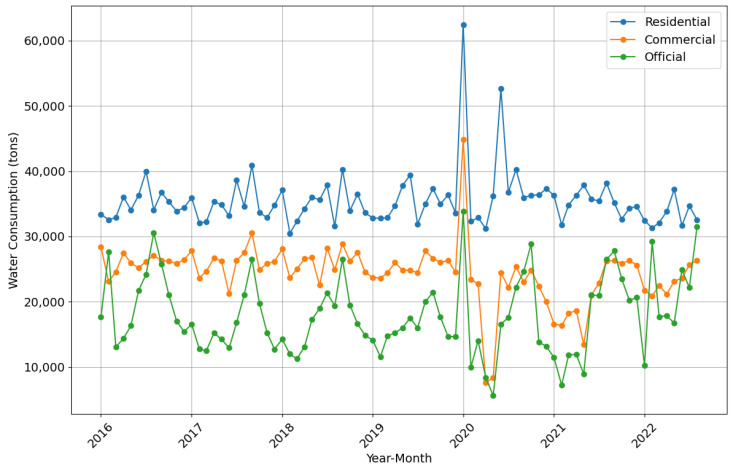
Monthly Water Consumption Trends.

Figure 2 illustrates monthly water consumption trends for residential, commercial, and official subscribers from January 2016 to August 2022. Commercial and official consumption reached their lowest points before mid-2020 (approximately February to May), coinciding with the onset of the COVID-19 pandemic. In contrast, residential consumption peaked during this same period, likely due to lockdown measures and increased time spent at home.

### 3.2. Key Features and Variables

The dataset includes the following key features and variables:

- Consumption Data: Monthly water consumption figures for each subscriber.
- Weather Parameters: Daily measurements of rainfall, sunshine, humidity, temperatures, and wind speed.
- Subscriber Details: Type of subscriber (residential, commercial, official), activity type, and tariff type.
- Temporal Information: Number of weekends and holidays in each month, and the phase of the COVID-19 pandemic.

Based on the correlation analysis and feature importance ranking, the most relevant features were selected: precipitation, wind_speed, sunshine_duration, max_humidity, min_humidity, max_temp, min_temp, saturday_sunday, holiday, household_size (residential), pandemy, prev4Month. These features were chosen due to their significant impact on water consumption patterns as identified in previous studies.

**Figure 3:**
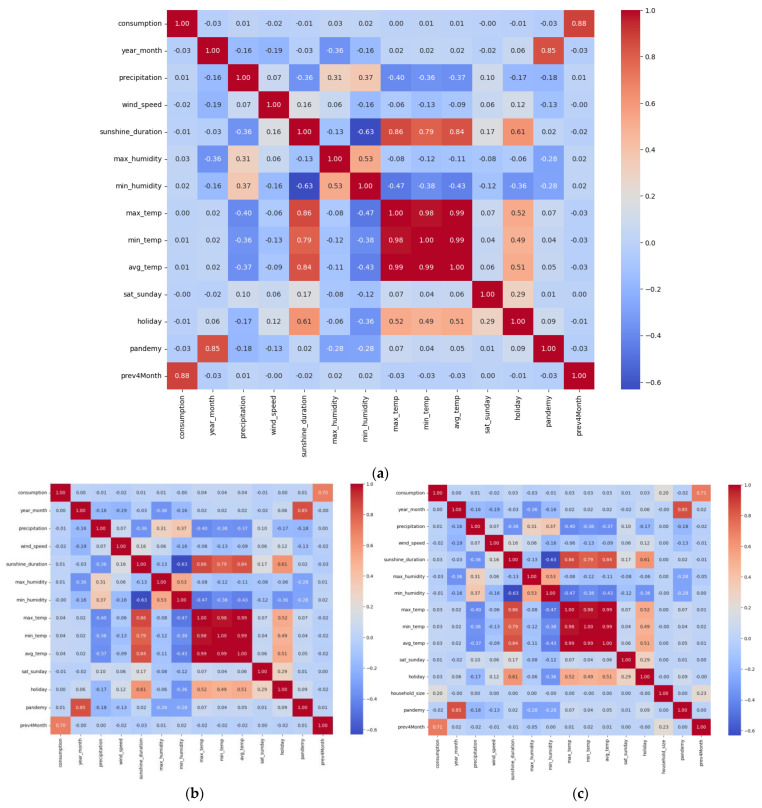
Correlation Matrices for (**a**) Commercial; (**b**) Official; (**c**) Residential.

Figure 3a–c presents the correlation matrices for the commercial, official, and residential datasets, respectively. These matrices illustrate the relationships between various features such as precipitation, temperature, and water consumption.

The correlation matrix of Commercial Subscribers in Figure 3a shows a weak positive correlation between consumption and precipitation (0.0062), indicating minimal direct impact. However, consumption has a strong positive correlation with historical consumption data (prev4Month: 0.8804), suggesting that past usage is a significant predictor. As shown in Figure 3b, similar patterns in the official data are observed with a weak negative correlation between consumption and precipitation (−0.0118) and a strong positive correlation with previous consumption data (prev4Month: 0.6968). The residential data shown in Figure 3c also has weak correlations between consumption and precipitation (0.0041) and significant positive correlation with historical consumption (prev4Month: 0.6972). These matrices highlight the importance of historical consumption data in predicting future usage across all subscriber types. The weak correlations with weather variables suggest that while these factors may influence consumption, their impact is less direct compared to historical usage patterns.

### 3.3. Data Preprocessing

#### 3.3.1. Handling Missing Values

Monthly water consumption data for 5000 subscribers were selected from over 800,000 based on their consistent subscription over an 80-month period, ensuring no missing values in their records. In contrast, the weather data contained significant missing values, which we addressed using the following methods:

- Spatial Interpolation: For locations with missing data, we calculated the arithmetic mean from neighboring locations to fill gaps, ensuring that imputed values reflected local weather conditions.
- Forward Fill Method: We applied forward filling to maintain temporal continuity in time series data, carrying forward the last known value for any gaps.
- Mean or Median Imputation: For any remaining missing values that could not be filled through the above methods, we used mean or median imputation based on the respective parameter.

By combining spatial interpolation, forward filling, and mean or median imputation, we ensured the dataset maintained both spatial and temporal integrity. This comprehensive approach maintains the essential methods and rationale for handling missing values, ensuring that the dataset retains both spatial and temporal integrity while enhancing clarity and efficiency.

Outlier detection was an essential part of the data preprocessing stage to ensure the accuracy and reliability of the predictions. We employed statistical methods, such as the Z-score method, to identify potential outliers in the dataset. Data points with Z-scores greater than 3 were considered outliers and were further examined to determine if they were due to measurement errors or genuine demand spikes. In cases where outliers were confirmed as measurement errors, they were corrected using interpolation methods based on surrounding data points. Genuine demand spikes, which were consistent with known events or patterns, were retained in the dataset to preserve the integrity of the real-world data.

#### 3.3.2. Normalization

Normalization ensures that all features contribute equally to the model training process by scaling them to a common range. The min-max normalization technique was applied to rescale the data between −1 and +1.

#### 3.3.3. Feature Selection

Feature selection involves identifying the most relevant features for the prediction task. This study used correlation analysis and feature importance ranking to select features that significantly impact water consumption. Correlation analysis was performed to assess the strength of the relationship between each feature and water consumption. Features with higher correlation coefficients were considered more relevant. Additionally, feature importance ranking was conducted using the Random Forest algorithm, which evaluates the contribution of each feature to the model’s predictive power. Based on these analyses, the most relevant features identified were precipitation, wind speed, sunshine duration, max humidity, min humidity, max temperature, min temperature, weekends, holidays, household size, pandemic period (COVID-19), and prev4Month. These features were chosen due to their significant impact on water consumption patterns, as identified in our analysis and corroborated by previous studies [33].

### 3.4. Machine Learning Techniques

All data processing and model training were performed using Python (Python Software Foundation, Beaverton, OR, USA) on the Google Colab platform (Google, Mountain View, CA, USA). The machine learning models (Artificial Neural Networks (ANN), Random Forest (RF), Support Vector Machines (SVM), and Gradient Boosting Machines (GBM)) and preprocessing techniques (e.g., scaling, feature selection) were implemented using the Scikit-learn library (Scikit-learn, Paris, France). The XGBoost algorithm was used with the XGBoost package (DMLC XGBoost, Python Software Foundation, Beaverton, OR, USA). Particle Swarm Optimization (PSO) was implemented via the Pyswarm library (Python Software Foundation, Beaverton, OR, USA), and deep learning models (LSTM) were developed using TensorFlow (Google, Mountain View, CA, USA).

The performance of each ML model was assessed using cross-validation and metrics such as R-squared (R^2^), Mean Squared Error (MSE), Root Mean Squared Error (RMSE), and Mean Absolute Error (MAE).

The selection of these specific machine learning models was based on their proven effectiveness in water consumption prediction and their ability to handle complex, non-linear relationships in time series data. ANN were chosen for their ability to capture intricate patterns and their successful application in previous water demand studies. RF was selected for its robustness to outliers and capability to handle high-dimensional data. SVM were included due to their effectiveness in handling non-linear relationships and their performance in similar environmental prediction tasks. GBM were chosen for their ability to improve prediction accuracy through ensemble learning. PSO and LM techniques were incorporated to explore the potential of optimization-based approaches in enhancing model performance. This diverse set of models allows for a comprehensive comparison of different machine learning paradigms in the context of water consumption prediction.

In this study, the RF model is an ensemble learning technique that constructs multiple decision trees to improve predictive accuracy. To enhance the performance of the RF model, we employed PSO to fine-tune its hyperparameters, resulting in the PSO Optimized RF model. This optimization process aims to identify the most effective combination of hyperparameters, such as the number of trees and their maximum depth. Additionally, the LM algorithm was used as an optimization method for training artificial neural networks, providing efficient convergence by combining gradient descent and the Gauss–Newton method.

### 3.5. Hyperparameter Tuning

In this study, hyperparameter tuning was conducted to optimize the performance of each machine learning model. For the ANN, we optimized the number of hidden layers, neurons per layer, activation functions, and learning rates. The optimal configuration was found to be two hidden layers with 64 and 32 neurons, ReLU activation, and a learning rate of 0.001. The RF model was tuned for the number of trees, maximum depth, and minimum samples split, resulting in an optimal configuration of 100 trees, a maximum depth of 10, and a minimum samples split of 2. For the SVM, the kernel type, regularization parameter (C), and gamma were optimized. GBM was fine-tuned for learning rate, number of boosting stages, and maximum depth. Additionally, PSO was used to optimize the number of trees and maximum depth for the RF model, and the LM algorithm was employed to optimize weights and biases in the Linear Model.

Training times varied, with SVM and GBM models being the most computationally intensive, taking over 12 h on our hardware setup (Intel Core i5, 16 GB RAM, 256 GB SSD (Intel, Santa Clara, CA, USA), NVIDIA GeForce RTX 3060 (Nvidia, Santa Clara, CA, USA)), while ANN and RF models were relatively faster, with training times under 2 h.

In our study, the hyperparameters of the RF model were determined using two distinct approaches: grid search and PSO [34,35]. Grid search is a traditional method that involves exhaustively searching through a specified subset of hyperparameters to find the optimal configuration. This method is straightforward but can be computationally expensive, especially with a large number of hyperparameters. On the other hand, PSO is a more advanced optimization technique inspired by the social behavior of birds and fish. PSO is used to efficiently explore the hyperparameter space by having a ‘swarm’ of candidate solutions (particles) that adjust their positions based on their own experience and that of their neighbors. This approach can often find optimal or near-optimal solutions more quickly than grid search, especially in complex hyperparameter spaces. 

These tuning techniques are crucial for enhancing the performance and accuracy of machine learning models, as evidenced by various studies that have demonstrated significant improvements in model outcomes through effective hyper parameter optimization [36,37].

### 3.6. Cross-Validation and Evaluation Metrics

Cross-validation was performed using K-Fold Cross-Validation (k = 5) to ensure model robustness and mitigate overfitting. In this method, the dataset is divided into five equal folds, where each fold is used once as a validation set while the remaining four serve as the training set. Evaluation metrics included R^2^, MSE, RMSE, and MAE.

### 3.7. Computational Efficiency and Feature Importance

In addition to predictive performance, the computational efficiency of the models and feature importance were analyzed.

The feature importance analysis reveals that historical consumption (prev4Month) is the most significant predictor across all subscriber types. Weather-related features such as temperature and humidity also play important roles. The impact of the COVID-19 pandemic (pandemy feature) is evident, indicating its overall influence on water consumption patterns.

This efficiency analysis is crucial for practical implementations where computational resources and time are limited. For real-time or near-real-time applications, faster models like ANN or RF might be preferred, while for offline analysis where prediction accuracy is paramount, the more computationally intensive models could be utilized.

Table 2 highlights the relative importance of different features in predicting water consumption across the merged dataset. Notably, ‘prev4Month’ consistently emerges as the most important feature, underscoring the significance of historical consumption patterns in forecasting future usage. Weather-related features, such as temperature and humidity, also play crucial roles, indicating their impact on water consumption trends.

Performance assessments of short-term water demand forecasting models have shown significant variations based on distinctive water uses, highlighting the need for tailored approaches [18].

To further illustrate the rationality of our feature selection, we conducted a comprehensive feature importance analysis. Table 2 presents the top 13 features ranked by their importance scores. This analysis provides strong evidence for the significance of our selected features in predicting water consumption.

The historical consumption data (prev4Month) emerged as the most crucial predictor with an importance score of 0.650643, far outweighing other features. This aligns with our correlation analysis and underscores the critical role of past consumption patterns in forecasting future water usage.

Weather-related features, including temperature (min_temp, avg_temp, max_temp) and humidity (min_humidity, max_humidity), showed substantial importance, with scores ranging from 0.011143 to 0.039674. This validates their inclusion in our model and highlights the significant impact of climatic conditions on water consumption.

Notably, the ‘pandemy’ feature, representing the COVID-19 pandemic period, ranked fourth in importance with a score of 0.025840. This confirms the pandemic’s considerable influence on water consumption patterns and justifies its inclusion in our predictive models.

Other features such as household_size, wind_speed, sunshine_duration, and precipitation also demonstrated meaningful importance, further supporting their selection for our models.

This comprehensive feature importance analysis, combined with our correlation study, provides a robust justification for our feature selection process. It demonstrates that our chosen features are indeed the most influential predictors of water consumption, ensuring the rationality and effectiveness of our modeling approach.

### 3.8. Data Splitting for Model Training and Testing

To ensure robust model evaluation, we employed a k-fold cross-validation approach for splitting our dataset into training and test sets. Specifically, we used 5-fold cross-validation, where the dataset was divided into 5 equal parts. In each iteration, 4 parts (80% of the data) were used for training, and 1 part (20% of the data) was used for testing. This process was repeated 5 times, with each part serving as the test set once.

This approach offers several advantages:

- It ensures that each data point is used for both training and testing, providing a more comprehensive evaluation of the model’s performance.
- It helps mitigate the impact of data variability and reduces the risk of overfitting.
- It provides a more reliable estimate of the model’s performance on unseen data.

The data splitting was performed randomly, but we ensured that the distribution of subscriber types (residential, commercial, and official) was maintained in both the training and test sets to avoid bias. Additionally, we maintained the temporal order of the data within each fold to preserve any time-dependent patterns in water consumption. For the final model evaluation reported in our results, we averaged the performance metrics across all 5 folds to obtain a robust estimate of each model’s predictive capability.

## 4. Results and Discussion

This section presents the results of the comparative analysis of the machine learning models used for water consumption prediction. The performance of each model is evaluated based on the selected evaluation metrics. The results are then discussed in the context of previous research and the specific conditions of the Kocaeli Province.

### 4.1. Model Performance

The performance of the six machine learning models (ANN, RF, SVM, GBM, PSO, and LM) was evaluated using the dataset described in Section 3. The evaluation metrics included R^2^, MSE, RMSE, and MAE. The results are summarized in Table 3.

As seen in Table 3, the GBM model achieved the highest R^2^ value of 0.881, indicating the best performance among the six models in terms of capturing the variability in the data. The GBM model also had the lowest MSE, RMSE, and MAE values, further confirming its superior performance.

It is important to note that Table 3 presents the overall performance metrics for each model across all subscriber types and time periods. To provide a more nuanced understanding of model performance, particularly in relation to different subscriber types and the impact of the COVID-19 pandemic, we conducted a more detailed analysis. Table 4 presents the performance of the GBM model, which showed the best overall performance, across different subscriber types and pandemic periods.

As shown in Table 4, the GBM model demonstrates consistent performance across different subscriber types and pandemic periods. For residential subscribers, the model maintains an R^2^ value above 0.28 across all periods, indicating a relatively stable predictive power. The performance for commercial subscribers, while lower, remains relatively consistent across the pandemic periods. For official subscribers, the model shows strong performance, particularly in the pre- and post-pandemic periods.

These results provide a more detailed view of the GBM model’s performance, highlighting its adaptability to different subscriber types and its resilience in the face of significant disruptions like the COVID-19 pandemic. The variation in performance across subscriber types suggests that tailored approaches may be beneficial for different categories of water consumers.

### 4.2. Discussion of Results

The results of this study highlight the effectiveness of different machine learning models in predicting water consumption. The GBM model, in particular, demonstrated superior performance across all evaluation metrics, suggesting that it is well-suited for this type of prediction task. The superior performance of the GBM model underscores its ability to capture complex, non-linear relationships in water consumption data, making it a valuable tool for water resource managers and policymakers. The GBM model demonstrated superior performance in predicting water consumption, achieving the highest R^2^ value of 0.881 among the models evaluated. This indicates its strong capability in capturing underlying data patterns. The GBM’s effectiveness stems from its ability to handle complex feature interactions and manage non-linear relationships. Its performance underscores the significance of historical consumption data as a predictor and its integration of environmental and temporal variables, such as precipitation and pandemic effects. The adaptability of the GBM model to different subscriber types further emphasizes its versatility in water consumption prediction. This aligns with previous studies that have shown the robustness of GBM in handling complex datasets and capturing intricate patterns in the data [38,39].

The ANN and RF models also performed well, with R^2^ values of 0.853 and 0.872, respectively. These models are known for their ability to model non-linear relationships, which is crucial in predicting water consumption patterns.

**Figure 4:**
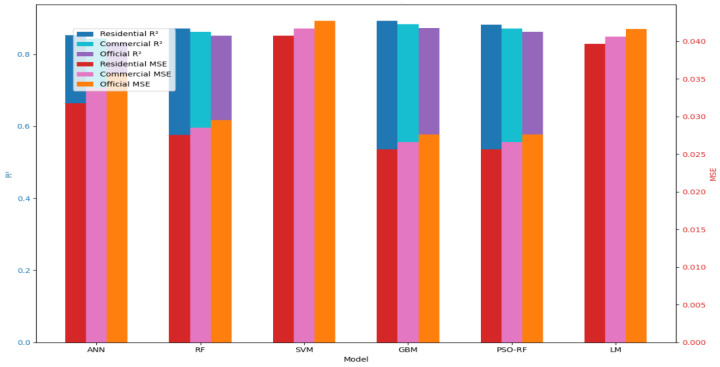
Comparative Performance of Models.

The SVM model, while slightly less accurate than the GBM, ANN, and RF models, still showed respectable performance with an R^2^ value of 0.809 (Figure 4).

The PSO and LM models, while not outperforming the GBM, still provided valuable insights into the prediction task. The PSO model, which incorporates optimization techniques, demonstrated an R^2^ value of 0.857, while the LM model, which focuses on second-order optimization, achieved an R^2^ value of 0.869. 

The PSO optimized RF model demonstrated improved performance over the standard RF model, achieving an R^2^ value of 0.872. The LM algorithm, a second-order optimization method, achieved an R^2^ value of 0.815, providing valuable insights into the prediction task. These results indicate that while optimization-based approaches can be effective, they may not always outperform well-tuned traditional machine learning models like GBM or RF.

**Figure 5:**
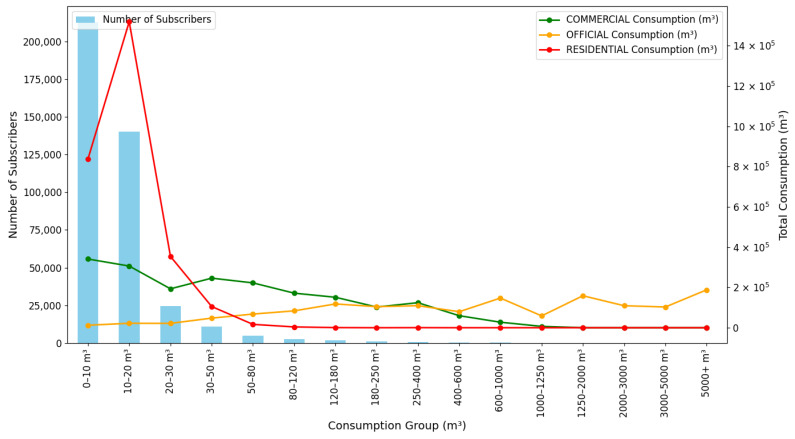
Distribution of Water Consumption Volumes and Total Consumption Across Groups by Subscriber Type.

To better understand the distribution of water consumption across different volume categories, we analyzed the data for all subscriber types. Figure 5 illustrates this distribution, showing both the number of subscribers and the total consumption for each consumption group.

The majority of subscribers fall into lower consumption categories, as indicated by the blue bars. However, the lines representing total consumption for each subscriber type reveal that higher consumption categories, despite having fewer subscribers, account for a significant portion of overall water usage. This is particularly evident for commercial and official subscribers, where consumption peaks in higher volume categories.

This analysis provides valuable insights for water management strategies. While the majority of subscribers are in lower consumption categories, the bulk of water usage comes from a smaller number of high-volume consumers. This suggests that targeted conservation efforts focusing on high-volume consumers could yield substantial water savings.

**Figure 6:**
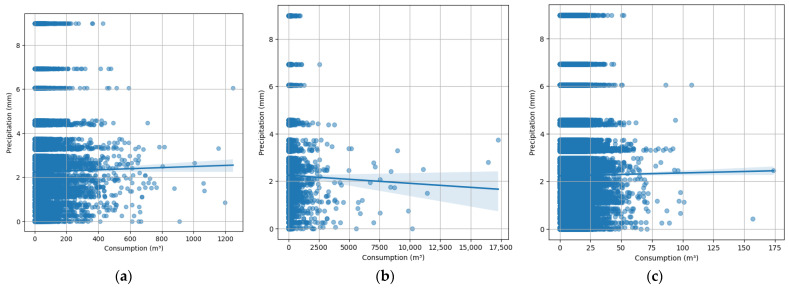
Scatter Plots of key 2-variable correlations for (**a**) commercial (m^3^); (**b**) official (m^3^); (**c**) residential (m^3^).

The scatter plots in Figure 6 highlight key two-variable correlations for different subscriber types, providing further insights into model performance.

The impact of the COVID-19 pandemic on water consumption patterns was also evident in the results. The models were able to capture the shifts in water usage during the pandemic, highlighting the importance of incorporating temporal data in prediction models.

Given that this study utilizes real-world data, deviations from trends reported in the literature may be observed. For instance, during the COVID-19 period, we might expect a rise in residential water consumption; however, a decrease could occur if residents temporarily relocated to other places. Similarly, while a reduction in water usage at commercial and official establishments might be anticipated, certain companies might show increased consumption due to specific operational policies or their area of expertise. Additionally, manual meter readings can introduce user errors, which should be factored into the analysis. This finding underscores the need for adaptive and resilient water management strategies that can respond to changing conditions [33,38].

### 4.3. Practical Implications

The findings of this study have several practical implications for water resource management in the Kocaeli Province. The superior performance of the GBM model suggests that it could be implemented in real-world applications to enhance the accuracy of water consumption predictions. This, in turn, can support more efficient water resource planning and management, helping to address issues such as water scarcity and leakage [20].

The comparative analysis also provides valuable insights into the strengths and limitations of different machine learning models. Practitioners can use these insights to select the most appropriate model for their specific needs, considering factors such as data availability, computational resources, and the complexity of the prediction task.

Moreover, the study highlights the importance of incorporating diverse features, including weather data and temporal variables, in prediction models. By leveraging a comprehensive dataset and advanced machine learning techniques, water resource managers can gain a deeper understanding of consumption patterns and develop more effective strategies for conservation and management [39].

**Figure 7:**
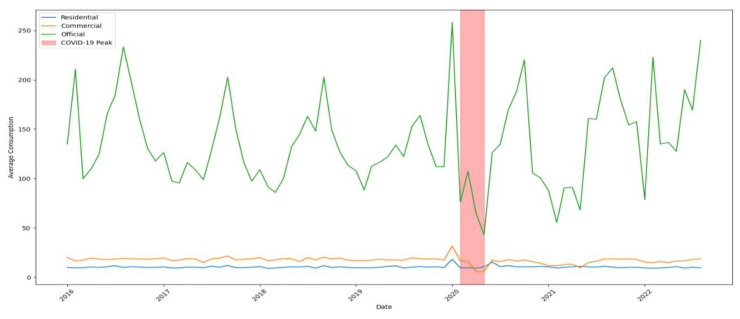
Impact of COVID-19 on Water Consumption by Subscriber Type.

The impact of the COVID-19 pandemic on water consumption patterns was also evident in the results. Figure 7 illustrates how water consumption varied across different subscriber types over time, with a noticeable change during the COVID-19 period marked in red. This highlights the importance of incorporating temporal data in prediction models to account for such significant disruptions.

### 4.4. Limitations and Future Research

While this study provides important insights into the application of machine learning models for water consumption prediction, there are several limitations that should be addressed in future research. First, the dataset used in this study is limited to the Kocaeli Province, and the findings may not be directly applicable to other regions with different climatic and socio-economic conditions. Future studies should consider applying these models to datasets from diverse regions to validate their generalizability.

Second, the study focuses on six machine learning models, but there are many other models and techniques that could be explored. Future research could investigate the performance of additional models, such as deep learning techniques, to further enhance prediction accuracy.

Lastly, the study highlights the impact of the COVID-19 pandemic on water consumption patterns, but it does not fully explore the long-term effects of such disruptions. Future research should consider longitudinal studies to examine how water consumption patterns evolve over time and in response to various external factors.

In conclusion, this study provides a comprehensive comparative analysis of machine learning models for water consumption prediction, with the GBM model emerging as the most effective. The findings offer valuable insights for water resource management and highlight several avenues for future research.

## 5. Conclusions

This study compared six machine learning techniques for water consumption prediction in Kocaeli Province, Turkey. The GBM model demonstrated the best performance with an R^2^ value of 0.881, followed closely by RF with 0.872. These results highlight the effectiveness of ensemble methods in capturing complex patterns in water consumption data.

Our analysis revealed that historical consumption data, weather parameters, and temporal factors significantly influence water consumption patterns. The impact of the COVID-19 pandemic was evident, with distinct consumption trends observed across different subscriber categories.

These findings have important implications for water resource management. By providing more accurate predictions of water consumption, these models can support proactive decision-making in water resource allocation, infrastructure planning, and conservation efforts. Water utility companies can leverage these models to improve demand forecasting and optimize resource allocation. Policymakers can use these insights to develop more targeted strategies for water conservation, particularly in regions facing water scarcity challenges like Turkey.

While this study provides insights, it is important to acknowledge its limitations, including the focus on a single region and the exclusion of water leakage data. Future research could explore ensemble models, incorporate additional data sources, and extend the analysis to multiple regions for broader applicability. Integration of IoT technologies for real-time data collection and more detailed socioeconomic data could enhance prediction accuracy and practical utility.

In conclusion, this study demonstrates the potential of machine learning techniques in water consumption prediction and underscores the importance of data-driven approaches in addressing water management challenges. As water scarcity continues to be a pressing global issue, such predictive models can play a crucial role in ensuring sustainable water resource management.

## Figures and Tables

**Table 1:** Summary of Dataset.

Data Type	Description
Water Consumption	Monthly data for 5000 subscribers (Residential: 3447, Commercial: 1422, Official: 131)
Weather Data	Rainfall, sunshine duration, temperatures, humidity, wind speed
Subscriber Info	Types (3), activity categories (132), tariff structures (20)
Temporal Data	Weekends, holidays, COVID-19 pandemic periods

**Table 2:** Top 13 Feature Importances.

Feature	Importance Score
prev4Month	0.650643
household_size	0.053207
min_temp	0.039674
pandemy	0.025840
min_humidity	0.021754
avg_temp	0.018872
max_temp	0.018281
wind_speed	0.016267
sunshine_duration	0.013252
max_humidity	0.011143
precipitation	0.010156
sat_sunday	0.006392
holiday	0.003765

**Table 3:** Model Performance Metrics.

Model	R^2^	MSE	RMSE	MAE
ANN	0.853	0.03178	0.1783	0.1231
RF	0.872	0.02754	0.1659	0.1145
SVM	0.809	0.04072	0.2018	0.1376
GBM	0.881	0.02563	0.1574	0.1095
PSO optimized RF	0.878	0.02563	0.1601	0.1132
LM	0.815	0.03964	0.1991	0.1354

**Table 4:** GBM Model Performance Across Subscriber Types and Pandemic Periods.

Subscriber Type	Pre-Pandemic (R^2^)	During-Pandemic (R^2^)	Post-Pandemic (R^2^)
Residential	0.318	0.316	0.280
Commercial	0.205	0.164	0.132
Official	0.616	0.439	0.607

## Data Availability

The data employed in this study were supplied by the Water and Sewage Service General Directorate of Kocaeli, Kocaeli, Turkey, a government institution. Due to the sensitive nature of the data, accessibility is limited, preventing its public dissemination. Nevertheless, the data can be made accessible upon reasonable request.

## Appendix A

These two tables present a sample of the original data used in the analysis for commercial, official, and residential subscribers. They include key features such as water consumption, weather parameters, and temporal data, providing insight into the structure and characteristics of the dataset.

**subscriber**	**subscriber_type**	**consumption**	**year_month**	**precipitation**	**wind_speed**	**sunshine_duration**	**max_humidity**	**min_humidity**	**max_temp**
10002	COMMERCIAL	15	201601	6.051	1.683	2.029	93.741	61.419	10.122
10002	COMMERCIAL	10	201602	2.786	1.575	3.351	91.793	52.896	16.448
10002	COMMERCIAL	13	201603	2.638	1.7	3.774	90.612	48.709	16.574
10005	COMMERCIAL	12	201611	2.593	1.476	3.373	93.066	53.066	16.996
10005	COMMERCIAL	16	201612	8.98	1.603	2.741	95.096	64.774	7.919
10003	OFFICIAL	7	201801	1941	1.512	2.658	93.967	59.225	12.116
10003	OFFICIAL	8	201802	2.071	1.728	1.721	93.928	64.642	13.025
10003	OFFICIAL	8	201803	3.316	1.838	3.796	94.29	52.935	17.758
10019	OFFICIAL	165	201810	1.838	1.093	3.619	89.29	55.935	22.148
10019	OFFICIAL	275	201811	1.886	1.456	1.946	91.366	63.4	16.33
1000	RESIDENTIAL	10	201601	6.051	1.683	2.029	93.741	61.419	10.122
1000	RESIDENTIAL	7	201602	2.786	1.575	3.351	91.793	52.896	16.448
1000	RESIDENTIAL	10	201603	2.638	1.7	3.774	90.612	48.709	16.574
1010	RESIDENTIAL	9	201711	1.853	1.416	3.473	94.066	58.2	17.526
1010	RESIDENTIAL	9	201712	4.561	1.348	3.196	90	55.129	15.925

**min_temp**	**avg_temp**	**sat_sunday**	**holiday**	**activity_type**	**tariff_type**	**meter_diameter**	**pandemy**	**prev4Month**	**household**
3.003	6.251	10	1	RESTAURANT	COMMERCIAL%50	DN20	0	15	x
7.472	11.358	8	0	RESTAURANT	COMMERCIAL%50	DN20	0	15	x
7.483	11.516	8	0	RESTAURANT	COMMERCIAL%50	DN20	0	12.5	x
8.23	11.870	8	0	BURO	COMMERCIAL%50	DN20	0	15.25	x
1.961	4.5	9	0	BURO	COMMERCIAL%50	DN20	0	16.5	x
4.829	7.845	8	1	OFFICIAL	OFFICIAL%50	DN20	0	6.75	x
6.025	9.082	8	0	OFFICIAL	OFFICIAL%50	DN20	0	7	x
8.022	12.148	9	0	OFFICIAL	OFFICIAL%50	DN20	0	7.25	x
14.674	17.58	8	1	HIGHSCHOOL	SCHOOL-HEALTH-SPORT%50	DN40	0	133.25	x
10.88	13.153	8	0	HIGHSCHOOL	SCHOOL-HEALTH-SPORT%50	DN40	0	98.75	x
3.003	6.251	10	1	FLAT	FLAT-1	DN20	0	10	3
7.472	11.358	8	0	FLAT	FLAT-1	DN20	0	10	3
7.483	11.516	8	0	FLAT	FLAT-1	DN20	0	8.5	3
9.08	12.61	8	0	FLAT	FLAT-1	DN20	0	7.5	2
7.706	11.07	10	0	FLAT	FLAT-1	DN20	0	8.25	2
